# University Experiences of Marine Science Research and Outreach Beyond the Classroom

**DOI:** 10.1093/icb/icab104

**Published:** 2021-05-27

**Authors:** Randi J Sims, Meghnaa Tallapragada, Tokea G Payton, Kara Noonan, Kathy L Prosser, Michael J Childress

**Affiliations:** 1 Department of Biological Sciences, Clemson University, Clemson, SC 29634, USA; 2 Department of Advertising and Public Relations, Klein College of Media and Communication, Temple University, Philadelphia, PA 19122, USA; 3 Educational Entertainment LLC, Seneca, SC 29679, USA

## Abstract

Climate and ocean literacy are two of the most important challenges facing society today. However, many students lack exposure to these topics upon entering college. As a result, these students must rely on learning climate literacy and ocean conservation through experiences outside of those provided in the traditional undergraduate classroom. To fill this gap, we initiated a marine science professional development program to expose undergraduate students to ocean literacy principles and climate change concepts through marine ecology research and educational outreach. This study evaluates the effects of our undergraduate experiential learning for individuals involved in our research team, our educational outreach team, or both. Clemson University alumni that participated in our program were surveyed to determine educational and professional gains in three areas related to: (1) knowledge; (2) careers; and (3) attitudes. Multiple linear and logistic regressions were used to understand the relationships between gains and program type, mentor experience, and duration of program enrollment. In addition, we evaluated demographic covariates including age, ideology, and gender. Our study found that perceived knowledge of marine science and science communication skills increased with positive mentor experience. Alumni that rated their experience with their mentors highly also indicated that the program was important to their careers after graduation. Students who participated in any program for a prolonged period were more likely to indicate that marine science was important to their careers. These students were also more likely to continue their education. Additionally, we saw that a sense of belonging and identity in science, as well as the understanding of climate change threat on the marine environment, all increased with longer program involvement, more than the type of experience (research versus outreach). Overall, we found that both the research and outreach programs offered opportunities for advancements in knowledge, careers, and attitudes. These results provide evidence that experiential learning has the potential to increase student engagement and understanding of climate change and ocean literacy communication as well as a sense of belonging in science-oriented fields.

## Introduction

Climate change has been especially detrimental to one of the most economically and biologically important ecosystems on Earth: the ocean (IPCC 2019). Problems such as rising temperatures, increased storm intensity, ocean acidification, and decreasing water quality are causing drastic declines to reef ecosystems (Harborne et al. 2017). Although the ocean is experiencing these issues, many students are not being exposed to climate change education (NCSE 2020) and ocean literacy (Gough 2017; Fauville 2019). The National Oceanic and Atmospheric Administration (NOAA) has attempted to fill this void through the creation of additional science standards centered on seven foundational ocean literacy principles, which address vital issues related to climate change and the marine ecosystem (NOAA 2020). However, many students in majors outside of marine biology have not been exposed to these principles, creating a need to integrate these topics outside of the traditional classrooms (Gould and Lewontin 1979; Gough 2017; Squarcina and Pecorelli 2017). Experiential learning is a widely accepted integrative concept used in modern educational techniques (Kolb and Kolb 2005). This “learning by doing” theory is based on the transaction of learning between students and their environment in which students contribute to their surroundings while their surroundings have internal impacts on them (Kolb 2015).

Experiential learning can be used in several formats but is commonly found in Undergraduate Research Experiences (UREs) and outreach experiences. UREs in Science, Technology, Engineering and Math (STEM) fields often place undergraduates into a research project alongside a faculty or graduate student mentor and are an effective way to give undergraduates their first encounters with biological inquiry and scientific communication (Nagda et al. 1998). Previous literature has found that participation in UREs can lead to increases in objective knowledge, perceived knowledge (confidence), science communication skills, and science identity, ultimately translating to more opportunities for advanced degrees, and a higher likelihood to graduate (Nagda et al. 1998; Bauer and Bennett 2003; Junge et al. 2010; Gilbert et al. 2014; Linn et al. 2015; Weaver et al. 2018). However, measured changes in personal attitudes and opinions on the research topic are not regularly presented in literature. Like UREs, outreach experiences can also lead to increases in perceived knowledge, science communication skills, and science identity (Rao et al. 2007; Bergerson et al. 2014; Carpenter 2015). Additionally, outreach programs have been shown to alter personal views toward pervasive issues (Bergerson et al. 2014; Carpenter 2015). Although these outcomes can be similar, outreach experiences in some instances integrate college undergraduates with K-12 students to begin conversations about pervasive scientific issues while teaching STEM concepts and principles. In return, this provides undergraduate students with a professional development opportunity that facilitates communication and mentorship with the next generation of students (Rao et al. 2007; Bergerson et al. 2014; Nelson et al. 2017; Knippenberg et al. 2020). While UREs provide undergraduates the opportunity to investigate and generate new ideas related to the field of interest, outreach experiences generally rely on conveying previously understood and well-defined topics to others (Kardash 2000; Rao et al. 2007; Junge et al. 2010; Wei and Woodin 2011; Carpenter 2015; Linn et al. 2015). Unfortunately, increases in objective content knowledge are not regularly measured in outreach programs. To the best of our knowledge, previous studies have not looked comparatively across research and outreach programs that address similar topics.

### Factors affecting gains in experiential learning

Both UREs and outreach experiential learning experiences primarily utilize the apprenticeship model to pair students with a graduate or faculty member to pursue a project (Nagda et al. 1998; Rao et al. 2007; Junge et al. 2010; Wei and Woodin 2011; Auchincloss et al. 2014). The relationships between mentors and undergraduate students have been shown as one of the most vital components of a successful college experience for undergraduates (Nagda et al. 1998). Students who are paired with mentors that emphasize career success and direction are more likely to overcome achievement gaps and find career success (Martin et al. 2013; Linn et al. 2015). Programs like UREs and outreach can also help to fill gaps in mentorships that many students experience upon entering college (Robnett et al. 2018).

Much like mentorship, length of experience can have profound effects on the success of these programs (Bauer and Bennett 2003). In an analysis of over 60 different UREs, one study found that the first year of involvement in the program led to almost no gains in identity, self-efficacy, concept retention, or relevant science skills (Linn et al. 2015). However, the longer the participants were enrolled, the more gains were seen in all areas (Linn et al. 2015). Similar results were found in a study conducted on undergraduates participating in K-8 STEM outreach (Nelson et al. 2017). Additionally, a study by Adedokun et al. (2014) found that students who participated in their summer URE found large gains in research skills and self-efficacy when undergraduates were enrolled for longer periods of time. Other studies have not used this as a focal point as this can be a difficult metric to measure in programs with a set length of enrollment (Junge et al. 2010; Lynch et al. 2010; Carpenter 2015).

### Creative inquiry program—Clemson University experiential learning

Clemson University, South Carolina, provides a creative inquiry program beyond the traditional classroom called that allows undergraduates to partner with graduate and faculty mentors on a broad range of experiential research projects. Many undergraduates have the freedom to rotate between creative inquiry teams as their interests evolve, or as a method to diversify their skillsets; an aspect that is unique to this program. Faculty receives modest grants to support the activities of the team. Most importantly, many of these programs encourage students to enroll for multiple semesters with the end goals being publications, presentations, research grants, and/or patents.

**Fig. 1.**
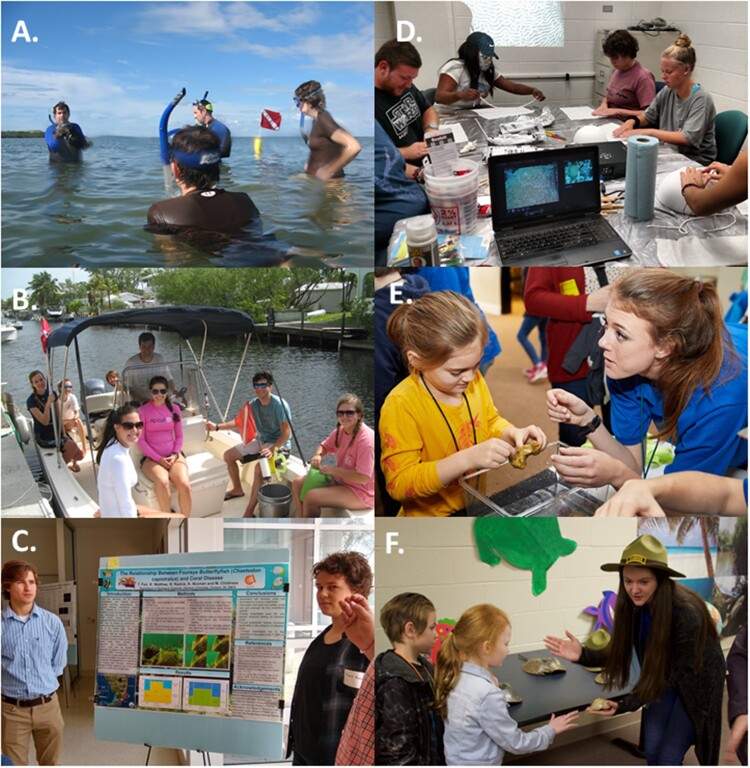
CMR creative inquiry team (**A**) learning marine species identification, (**B**) conducting marine ecology research, (**C**) presenting research findings at a University Symposium. SVF creative inquiry team, (**D**) building coral reef theatrical set, (**E**) marine veterinarian sharing live invertebrates, (**F**) park ranger exploring sea turtle nesting beach. Photo credits with permission to: (**A**) Pete Bouwma, (**B**) Kylie Smith, (**C–D**) Michael Childress, (**E–F**) Robert Bradley. All permissions obtained.

The Conservation of Marine Resources (CMR) creative inquiry team is focused on marine and behavioral ecology field research exploring the impacts of climate change and habitat loss on the behavior and ecology of marine invertebrates and reef fishes (Fig. 1A–C). CMR is only advertised to students through their professors and academic advisors. Students must inquire and apply to CMR by submitting a personal statement and curriculum vitae. This creative inquiry primarily attracts students majoring in Biological Science, Animal Veterinary Science, Environmental Science, Wildlife and Fisheries Biology, and Biosystems Engineering. Applicants are then interviewed and selected on a competitive basis determined through GPA, research and animal care experience, and overall interest in one of our ongoing projects. Once accepted, all students that are approved by their mentor are invited to continue in the program until they graduate. Students in CMR learn various methods of quantifying species abundances and behaviors using imaging software and statistical analysis programs. Those team members with open-waterSCUBA certifications may also participate in the data collection in the field during the summer semesters. All students are required to participate in weekly scientific paper discussion groups on current topics in marine science and partake in science communication involving either a poster or oral presentation at a university, regional, or national conference during the school year. Some students are also given the opportunities to aid in the publication process based on their skills and interests.

The Something Very Fishy (SVF) creative inquiry team is a marine science educational outreach team focused on teaching the principles of climate and ocean literacy to elementary students (Fig. 1D–F). SVF is actively advertised through the Creative Inquiry program, and through multiple departmental email lists. This creative inquiry attracts students majoring in Biological Science, Animal Veterinary Science, Environmental Science, Education, Psychology, and Wildlife and Fisheries Biology. Any student who inquires about joining SVF is immediately cleared to enroll. Students who participate in the program by attending weekly meetings and the STEAM exhibits are automatically invited to continue until graduation. Involvement in this outreach program includes a Broadway style musical theatre performance followed by various science exhibits to help educate elementary school students about ocean conservation. SVF undergraduate students learn about threats to ocean health, introductory marine science, climate change threats, learning styles, and storytelling concepts through lectures, group discussions, and the creation of learning modules. These students are also responsible for the development of interactive exhibits where they portray different careers in science (coral biology, marine animal veterinarian, park ranger, SCUBA engineer, sea turtle biologist, etc.) as docents on an imaginary field trip to the Florida Keys. The STEAM exhibits seek to combine arts and scientific approaches in teaching ocean literacy principles. After the children attend the *Something Very Fishy* musical theatre production, a partner of the Clemson SVF creative inquiry team, the SVF undergraduate students teach the elementary school students about ocean conservation while portraying a career in science.

Here we compare students that have participated in two different creative inquiry teams, CMR (2008–2020) and SVF (2018–2020). Previous studies have found alumni to be an accurate representation of undergraduate perceptions on URE’s (Adhikari and Nolan 2002). Alumni are also more likely to understand how the program impacted their career, personal gains, and attitudes, as well as concept retention (Junge et al. 2010). Thus, our study uses CMR and SVF Creative Inquiry alumni to measure the gains of our programs on undergraduates. This study aims to understand the unique gains for undergraduate students of a marine biology outreach experience versus a marine biology research experience, versus students that experienced both. Success in these experiences is determined through three metrics: (1) knowledge; (2) career; and (3) attitudes (Table 1). These gains are also compared to length of involvement (duration) and mentorship experience in the type of creative inquiry program (research versus outreach versus both).

**Table 1:** Dependent variables affected by program involvement in categories related to our three gains: knowledge, careers, and attitudes

Knowledge	Careers	Attitudes
Objective knowledge of ocean literacy concepts	Importance of marine science on career	Science identity and belonging
Perceived knowledge of marine science	Importance of the program on graduation	Perception of climate change threat on marine environment
Marine science resource skills	Pursuance of STEM career	Importance of conservation on daily life
Marine science stewardship skills	Pursuance of further education	—
Marine science communication skills	—	—

## Methods

### Undergraduate student survey

A 35 question Qualtrics survey was created to collect information regarding our independent variables (duration in the program, type of program, and mentorship experience), our dependent variables (knowledge, career, and attitudes), and several potential demographic covariates (age, gender, ethnicity, and ideology). Our survey questions were approved by the Clemson University Institutional Review Board (IRB2018-497) and are shared in the Supplementary Tables S1–S5. Survey invitations were sent by e-mail from the program leads to all alumni of our SVF outreach and CMR research creative inquiry teams, including those who had participated in both. All respondents were given instructions on how to access the survey and were assured anonymity in their responses. We sent surveys to a total of 121 alumni, 71 who participated in our outreach program, 37 in our research program, and 13 from both. Respondents answered questions regarding mentor experience and overall experience for the program(s) they were involved in. Students who were involved in both programs answered all questions. Alumni in outreach were also asked what roles they filled during their participation, while previous research students were asked what projects they were involved in. Respondents who participated in both were asked both sets of questions. All respondents were asked questions related to their gender, age, and political affiliation as well (Supplementary Table S2).

### Survey measures

Alumni of all classifications were asked to indicate their perceived knowledge of marine science concepts on a scale of 1–5 (1 = nothing at all and 5 = expert-level) (Supplementary Table S3). Because an ocean literate person is defined as someone familiar with the seven ocean literacy principles, respondents were given each principle and asked to indicate how true they felt each was on a four-point scale (1 = Definitely False and 4 = Definitely True) (NOAA 2020). Alumni were also asked to indicate if their program involvement influenced their communication, research, and stewardship skills (1 = Strongly Disagree and 5 = Strongly Agree). Career choice was broken up by STEM careers (medical, research, or other career entered by text related to a STEM career), continuing student, or other (Supplementary Table S4). These responses were recorded as binary variables. Respondents were asked if various skills learned in their program assisted them in their career. These skills included: communication, research, and conservation strategies which were rated on a scale of 1–5 (1 = strongly disagree and 5 = strongly agree). Finally, respondents were asked to identify the importance of marine science overall in their current career fields on a scale of 1–5 (1 = Not at all important and 5 = Extremely important). Science identity and belongings were assessed through several statements related to attitude (Supplementary Table S5). These statements included the importance of discussing new ideas in science, the value of research, the ability of science to solve problems, and the feeling of discovery as “thrilling.” Responses were rated on a scale of 1–4 (1 = Not at all like me and 4 = Very much like me). Belonging in science was assessed through respondents’ answers to direct statements on belonging in a science-related field. Statements assessing willingness to communicate about science to the public were also used to indicate belonging. All statements were rated on a five-point scale (1 = Strongly disagree and 5 = Strongly agree). Alumni were asked to rate their perception of critical ocean health issues on a scale of 1–4 (1 = No threat at all and 4 = High threat). Finally, respondents were asked to rate how important conservation was on their daily lives (1 = Not at all important and 5 = Very Important). All semesters that a respondent participated in any program were summed to calculate total duration of involvement. Program participation was broken down by category: outreach, research, or both. Mentor experience was determined by averaging questions related to mentor experience (Supplementary Table S1). All scales were found to be reliable (see Supplementary Materials for more details).

### Statistical analyses

We used a multiple model comparison approach (Burnham and Anderson 2002) to evaluate which factors were most important for each of our dependent variables. Our multiple linear regression models always included our three demographic covariates: gender (female only due to our demographic distribution), age, and ideology (very conservative to very liberal). Each model then included from zero to four independent variable terms; semesters enrolled in creative inquiry (duration), ratings of CI team leader mentorship (mentor), and creative inquiry type (CMR, SVF, or both). All possible combinations of independent variables were evaluated and compared using a minimum AICc ranking (Supplementary Table S6). Best fit models were defined as those with _**Δ**_AIC scores of less than two (Table 2). Because the “pursuance of further education” and “pursuance of a STEM career” were binary dependent variables, we used logistic multiple regressions with a binomial distribution.

**Table 2:** Multiple linear regressions and logistic regressions (indicated by an asterisk) with AICc scores <2

Knowledge										
	Age	Gender	Ideology	Duration	Mentor	Research	Outreach	Overall model		
	*β*1	*β*2	*β*3	*β*4	*β*5	*β*6	*β*7	F	*P*	Adj. *R*^2^
Perceived knowledge of marine science	0.0066	0.1293	0.0395	*0.1014*	**0.5824**	—	—	2.8304	0.0369	0.2337
	−0.0148	−0.0429	0.0473	—	**0.7002**	—	—	2.2155	0.0951	0.1394
Marine science communication skills	0.0189	−0.0075	0.0570	*0.0785*	**0.7357**	—	—	3.8940	0.0095	0.3253
	0.0023	−0.1409	0.0630	—	**0.8269**	—	—	3.7663	0.0152	0.2694

**Careers**										

	**Age**	**Gender**	**Ideology**	**Duration**	**Mentor**	**Research**	**Outreach**	**Overall model**		
	*β*1	*β*2	*β*3	*β*4	*β*5	*β*6	*β*7	F	*P*	Adj. *R*^2^
Importance of marine science on career	*−0.1258*	−0.1329	0.0463	**0.2164**	—	—	—	3.8630	0.0136	0.2762
	−0.1082	−0.0212	0.1314	**0.1929**	0.5809	—	—	3.4084	0.0175	0.2864
Importance of the program after graduation	−0.0468	0.4967	0.1511	—	**0.6629**	—	—	3.1520	0.0307	0.2230
	**Age**	**Gender**	**Ideology**	**Duration**	**Mentor**	**Research**	**Outreach**	**Overall model**		
	*β*1	*β*2	*β*3	*β*4	*β*5	*β*6	*β*7	c^2^	*P*	
Pursuance of further education*	−*1.0985*	−1.2829	−0.1430	*0.4276*	—	—	—	18.657	0.0009	
	−*1.3261*	−1.8268	−0.6023	**0.5447**	−2.3397	—	—	20.855	0.0009	
	**−0.7826**	−1.8452	−0.2492	—	—	—	—	14.341	0.0025	

**Attitudes**										

	**Age**	**Gender**	**Ideology**	**Duration**	**Mentor**	**Research**	**Outreach**	**Overall model**		
	*β*1	*β*2	*β*3	*β*4	*β*5	*β*6	*β*7	F	*P*	Adj. *R*^2^
Science identity and belonging	**−0.0451**	0.0071	0.0218	**0.0685**	—	—	—	4.5380	0.0065	0.3205
Perception of climate change threat on the marine environment	0.0270	0.1244	***0.1226***	**0.0515**	—	−0.2261	0.1809	4.0953	0.0057	0.3823
	−0.0109	0.1757	***0.1185***	*0.0349*	—	—	—	3.7887	0.0148	0.2710
	−0.0199	0.1054	***0.1150***	—	—	—	—	3.6885	0.0240	0.2118
	0.0152	0.0397	***0.1132***	—	—	**−0.3488**	−0.0382	3.4501	0.0166	0.2899

These show the multiple models run for each dependent variable and its category. Individual factors significant in each model are indicated in italics (*P* < 0.10), bold (*P* < 0.05), and bold italic (*P* < 0.01).

## Results

### Demographics

We received a total of 37 responses for a 31% participation rate, notably higher than Clemson University alumni’s normal response rate of <10%. Alumni were asked to identify which program they participated in (11 in outreach, 15 in research, and 11 in both) as well as their length of participation (Supplementary Table S1). Respondents that failed one or more attention checks were removed from the dataset to ensure accurate answers, this resulted in a total of 31 usable responses from alumni who participated in our programs (*N* = 9 in outreach, *N* = 13 in research, and *N* = 9 in both). Participants ranged in age from 20 to 33 years, with the majority between 20 and 26 (*M* = 24, SD = 3.58). Most of our subjects identified as female (*N* = 25), with only five males and one who preferred not to disclose their gender. This is representative of gender distribution in both our program and across majors who participate in our program (Supplementary Table S7). Our sample consisted mainly of Caucasian alumni (*N* = 29) with only two alumni identifying as part of a minority group (Black or African American *N* = 1; Asian *N* = 1). These numbers are in alignment with university demographics as well as demographics across majors who participate in our program and our program is representative of both of our programs’ demographics (Supplementary Table S7). We defined ideology as the political views held by our alumni, this ranged from (1) Very Conservative to (5) Very Liberal. While we had a range of responses regarding political ideology, the majority identified as somewhat to very liberal (*M* = 3.58, SD = 1.26).

### Knowledge

Perceived knowledge of marine concepts was influenced the most by a positive mentor experience (**_*β*_ **= 0.5824, *P* = 0.0375) and secondarily by time in the creative inquiry team (**_*β*_ **= 0.1007, *P* = 0.0511). However, the creative inquiry type did not influence perceived knowledge, nor did any of the demographic covariates (Table 2). Marine communication skills were influenced the most by a positive mentor experience (**_*β*_ **= 0.7357, *P* = 0.0047) and secondarily by time in the creative inquiry team (**_*β*_ **= 0.0785, *P* = 0.0878). Like perceived knowledge, the creative inquiry type did not influence marine communication skills, nor did any of the demographic covariates (Table 2). We did not find any relation between creative inquiry type, duration, or mentor experience on stewardship or resource skills. Objective knowledge of ocean literacy principles was also not related to creative inquiry type, duration, or mentor experience. However, the knowledge of ocean literacy principles was relatively high in all creative inquiry teams: outreach (*M* = 3.53, SD = 0.31), research (*M* = 3.51, SD = 0.24), and both (*M* = 3.51, SD = 0.22).

### Career

Alumni respondents primarily held careers in the STEM category (*N* = 12) or were continuing students in a science field (*N* = 12) with only seven holding jobs in communication or another field. The importance of marine science on career was primarily influenced by the time in creative inquiry (**_*β*_ **= 0.2164, *P* = 0.0252) and secondarily by age (**_*β*_ **= −0.1258, *P* = 0.0797). The influence of alumni age indicated that the longer they had been out of the program, the less important marine science was on their careers. Neither mentorship, creative inquiry type, gender, nor ideology influenced on the importance of marine science on their career (Table 2). However, the importance of the program after graduation was primarily influenced by a positive mentorship (**_*β*_ **= 0.6629, *P* = 0.0155), with those who had more positive mentor experiences finding the program more important after they graduated (Table 2). Those who were younger (log regression _**χ**_^2^ = −1.098, *P* = 0.0535) or involved in creative inquiry for many semesters (log regression _**χ**_^2^ = 0.4276, *P* = 0.0729) were more likely to continue their education (Table 2). All students who indicated that they were continuing students, also stated that it was in a STEM field. There was also no significant effect of creative inquiry type on the importance of the skills learned in the program on the alumni’s careers.

### Attitudes

A sense of science identity and belonging increased primarily with time in creative inquiry (**_*β*_ **= 0.0685, *P* = 0.0196) and decreased secondarily with age (**_*β*_ **= −0.0451, *P* = 0.0396). Interestingly, those who were younger had a stronger sense of science identity and belonging. Neither mentorship nor creative inquiry type influenced science identity and belonging (Table 2). Students who participated in both programs were more likely to have higher perceptions of threats to the ocean’s health versus those who only participated in the research program (**_*β*_ **= −0.3488, *P* = 0.0413). However, ideology was an even stronger driver in these perceptions of threat (**_*β*_ **= 0.1132, *P* = 0.0074) with very liberal alumni showing higher levels of perceived threat (Table 2). The importance of conservation in daily life was not impacted by creative inquiry type, duration, or mentorship experience.

### Overall experience

It is worth noting that alumni from both CMR and SVF felt positive about their experience in their respective creative inquiry teams (research: *M* = 4.54, SD = 0.52; outreach: *M* = 4.44, SD = 1.67; both *M* = 4.83, SD = 0.25). This was also true for mentorship experience (research: *M* = 4.55, 0.64; outreach: *M* = 4.67, SD = 0.55; both: *M* = 4.80, SD = 0.33). Respondents also indicated strong positive feelings toward their program(s) through the additional comments left at the end of the survey (Supplementary Table S8).

## Discussion

The purpose of this study was to determine the effects of both outreach and research creative inquiry on undergraduate alumni in relation to marine and climate change knowledge and skills, career choice, and attitudes. We found evidence that creative inquiry impacted perceived knowledge and skills significantly through mentorship experience, but this did not depend on the creative inquiry type (research versus outreach) or duration. Career choices and factors related to career choices were impacted by duration and mentorship experience, but not creative inquiry type. We also found that alumni indicated a higher sense of science identity and belonging the longer they were enrolled in creative inquiry as well as better understanding of threat perception, which was also influence by personal ideology. Creative inquiry type influenced attitudes toward ocean threats.

### Knowledge

In both outreach and research creative inquiry, graduate mentors, and a primary faculty advisor partner with students in small groups or individually. We found that perceived knowledge of marine science was higher in alumni who rated their experience with their mentors highly. We also observed gains through positive mentorship in one area of science that is not typically addressed in lecture-style STEM classes: communication. In the modern field of marine science, communication is one of the most critical skills not taught beyond the general education requirement in Universities (Gill and Golding 2001). Students within both programs engage in various forms of science communication including poster presentations, science exhibit facilitation, oral presentations, blog posts, and discussion leadership. These presentations are normally given and subsequently critiqued by their mentors before presentation to a general audience. Working closely with mentors to understand the concepts and hypotheses they are presenting allows students to simultaneously increase their confidence in their research while promoting strong communication (Kardash 2000; Linn et al. 2015). Our findings were similar to other studies who found that good experiences with mentors also lead to higher self-efficacy and confidence, particularly in STEM fields (Kardash 2000; Lopatto 2007). Students with higher self-efficacy in science become more engaged with science and have higher retention rates, as well as a better understanding of the discipline overall (Andrew 1998; Sawtelle et al. 2012; Macphee et al. 2013; Williams and George-Jackson 2014). This can be particularly impactful to those in underrepresented groups as well as women (Macphee et al. 2013; Ballen et al. 2017). Similar gains were seen through longer involvement with the program. Because our students could potentially enroll anywhere from one semester to all 4 years of their college career in either or both programs, students generally become involved in multiple projects, increasing their exposure to and confidence in different subject areas of marine biology. This lends way to more opportunities for scientific communication and facilitates their ability to hone their craft over time (Carpenter 2015). These findings have numerous implications for students’ future motivations in science and gives evidence to the successful nature of our programs.

### Careers

Mentors throughout all parts of a student’s undergraduate experience can have significant influences on the skills they acquire in college, their desire to continue their higher education, and ultimately the career they choose to pursue (Houser et al. 2013; Langholz and Abeles 2014). Students in our creative inquiry teams who felt encouraged and heard by their mentors believed that their program involvement was critical to their success after graduation. Most of our alumni also indicated that they were involved in a STEM field whether it was continuing education or as a career. Those that were involved in the program for longer durations were more likely to continue their education, although age was also a contributing factor. This suggests that those involved in the program for longer are more likely to continue their education, particularly when they are between the ages of 20–26 years. This is not an atypical finding as other programs have found that long-term involvement, or greater involvement with a project can lead to higher immediate motivation to pursue graduate school (Russell et al. 2007; Linn et al. 2015). Because many of our participants were between the ages of 20–26 years, further research is needed to evaluate the potential impacts that time spent in this program has on older alumni who are more advanced in their careers.

### Attitudes

The perception of climate change threats on ocean health was higher for those who participated in both programs than research alone. Students are taught directed lessons on climate change threats in the outreach program and later communicate them to elementary students. Those that participate in research later couple this primary knowledge with research projects that attempt to combat the threats of climate change. Techniques like this that connect students directly to the ocean have the potential to contribute to undergraduates’ understanding of climate change education (Gough 2017; Squarcina and Pecorelli 2017). Thus, pushing to involve undergraduates in community ocean literacy outreach, particularly at research-centered universities could be beneficial for increasing climate change awareness in both undergraduates and the public (Plankis and Marrero 2010; Visbeck 2018). It is also important to point out that ideology was one of the strongest significant negative factors in all models. Previous literature has found that political ideology can influence the perception that many students have on perceived risks of other controversial scientific topics (Ferguson et al. 2020). Other studies have also found that by incorporating previously held values and beliefs into educational platforms, one is more likely to be accepting of scientific concepts and understand these concepts better (Miyake et al. 2010; Corner et al. 2015). While this does not negate the important impact of program type in our study, it does provide insight into the extreme influence that personal beliefs can have on one’s perception of climate change threats (Lawson et al. 2019). This also leads to the suggestion that when introducing these topics to any audience, careful consideration must be taken to incorporate their intrinsic values and beliefs into the lesson.

One of the most important parts of experiential learning is the effect it has on a student’s feeling of belonging and identity (Kolb and Kolb 2009). In our study, students who were enrolled for longer periods of time felt more like they belonged in science and that they identified as a scientist. This is extremely encouraging as a student’s positive relationship with science can encourage them to continue their scientific pursuits (Carlone and Johnson 2007). Research has shown that first-generation college students and underrepresented minority groups that traditionally have a harder time developing their science identity could benefit greatly from participating in experiential learning (Linn et al. 2015; Jackson et al. 2016). Unfortunately, our study did not have a large diversity in ethnic groups, but this study provides further evidence that both outreach and UREs can assist students in developing their scientific identity and sense of belonging with time and effort.

## Limitations and future directions

While the use of alumni provides valuable insight in understanding the lasting impacts an undergraduate creative inquiry may have on student success, it can be difficult to attribute success metrics to the creative inquiry alone. This is a particular problem in our creative inquiry teams as students may come in with a wide range of previous knowledge about marine science and climate change. While we attempted to control for factors such as ideology, age, and gender, it cannot be ignored that we did find some evidence of the effect that both age and ideology had on gains. This is particularly true for variables such as threat perception, where ideology was significant regardless of the independent variable. Our small sample size could also have resulted in having low power to detect an effect or possibly inflate some effect sizes. We encourage scholars to replicate our research using appropriate sample sizes driven by *a priori* power analysis to help us better understand the effects of UREs and outreach programs on undergraduates. Because many of our students indicated that they felt positive about the program, those who felt negative may have disregarded survey requests. Therefore, we must consider that the results of this study may not be representative of all alumni experiences. Future studies should focus on using pre- and post-enrollment surveys to evaluate true quantitative gains throughout the course of the programs. This is currently being implemented in our outreach creative inquiry. We are also planning to continually monitor program success through periodic alumni surveys. Control groups are also a general source of contention in these types of studies. It can be especially difficult to find a comparable group to that of outreach or research programs as these are normally competitive programs, or programs that focus on unique concepts outside of the normal curriculum. Future research should consider comparing these types of programs against those enrolled in a traditional lecture-style course on the subject. Finally, while our study demographics were overall comparable to our university and majors that participated in our program, these demographics are not necessarily reflective of other institutions or their departments. Therefore, careful consideration should be taken in extrapolating these findings to departments which may have a greater diversity of participants. We are currently exploring options to diversify our program in a meaningful way.

## Conclusion

Our study contributes to the growing body of literature suggesting the impact that experiential learning can have on student knowledge, careers, and attitudes. Although our creative inquiry teams were vastly different in their approaches, the messages across them both were the same: ocean conservation is important. Both exemplified using experiential learning to engage students within this important topic. While we initially sought to look comparatively at gains across both research and outreach teams, our findings suggest that both forms of experiential learning can be used as a tool to increase perceived knowledge, communication skills, conservation desire, and lead to a higher sense of science identity and belonging. As shown in our study, educators interested in integrating experiential learning into their curriculum should consider creating long-term opportunities for their students while providing open collaboration with mentors. Employers of experiential learning should also account for student values and experiences such as ideology, when designing a research, or outreach program. Although limitations such as funding, time, and faculty/graduate student involvement can inhibit the integration of experiential learning, this study shows the importance of experiential learning on undergraduate success. This study also provides evidence for experiential learning to combat deficits in climate change and ocean literacy knowledge. We encourage the continued use of such techniques to simultaneously contribute to young adult’s understanding of climate change while allowing them opportunities to combat it through research and outreach.

## Supplementary Material

icab104_Supplementary_Data

## Notes

“From the symposium “SICB-Wide Symposium: Biology beyond the classroom: Experiential learning through authentic research, design, and community engagement” presented at the virtual annual meeting of the Society for Integrative and Comparative Biology, January 3–7, 2021.”
