# Supplementary material for: University Experiences of Marine Science Research and Outreach Beyond the Classroom
Source: Integr Comp Biol. 2021 May 27;61(3):1078–88. doi: 10.1093/icb/icab104 (PMC8490691; doi:10.1093/icb/icab104)
Supplement: icab104_Supplementary_Data [file icab104_supplementary_data.docx]

**Supplemental Materials**

Single question continuous variables derived from a single response on a five-point scale: Ideology (Table S2), Perceived Knowledge of Marine Science (Table S3), Marine Science Communication Skill (Table S3), Marine Science Resource Skill (Table S3), Marine Science Stewardship Skill (Table S3), and Importance of Marine Science on Career (Table S4).

Multiple question continuous variables derived from the average responses on a five point scale: Mentor Experience (Table S1), Objective Knowledge of Ocean Literacy Concepts (Table S3), Importance of the Program on Graduation (Table S4), Science Identity and Belonging (Tables S5), and Perception of Climate Change Threat on Marine Environment (Table S5). All multiple questions variables had a Cronbach’s alpha > 0.80 to assure composite score reliability.

Two binary variables were constructed from a single multiple-choice survey question “*What is your current job/career field?”* (Table S4): Pursuance of STEM Career (Yes = Medicine, Research, Other STEM; No = Education, Communication, Industry, Not yet graduated, Graduate student, Other Non-STEM ); and Pursuance of Further Education (Yes = Graduate student, Not yet graduated; No = Education, Medical, Research, Communication, Industry, Other).

Linear and logistic multiple regression models (Table S6) were calculated in R 4.0.3 using the lm and glm (family=binomal) functions and confirmed in the fit model multiple regression procedure in JMP 14.1.0.

Table S1: Survey questions used to determine independent variables.

| **Program Type**  *Which Creative Inquiry(ies) have you participated in?* |
| --- |
| Something Very Fishy (Outreach) |
| Conservation of Marine Resources/Marine Ecology (Research) |
| Both |
| **Duration**  *Indicate all semesters you participated in the Conservation of Marine Resources or Marine Ecology Creative Inquiry and/or Something Very Fishy. Click all that apply.* |
| **Mentor Experience** (1 = Strongly Disagree to 5 = Strongly Agree)  *Thinking back to your experience with your Conservation of Marine Resources or Marine Ecology mentor(s) and/or Something Very Fishy mentor(s), please indicate your level of agreement with the following statements:* |
| My mentor(s) helped provide direction and guidance on professional issues |
| My mentor(s) acknowledged my contributions appropriately |
| My mentor(s) actively listened and provided useful critiques |
| My mentor(s) motivated me to improve my work |
| Overall, I was satisfied with my mentor(s)  *Cronbach’s alpha (Conservation of Marine Resources/Marine Ecology) = 0.93;Cronbach’s alpha (Something Very Fishy) = 0.92* |

Table S2: Survey questions related to demographics used as covariates in all models.

| **Age** (text entry)  *How old are you?* |
| --- |
| **Gender** (female)  *What is your gender?* |
| Male |
| Female |
| Non-binary, gender fluid, or gender non-conforming |
| Other (text) |
| Prefer not to disclose |
| **Ideology**  *Generally speaking, would you describe your political views as* |
| Very conservative |
| Somewhat conservative |
| Moderate |
| Somewhat liberal |
| Very liberal |

Table S3: Survey questions used to determine gains in conceptual understanding.

| **Objective Knowledge of Ocean Literacy Concepts** (1 = Definitely False to 4 = Definitely True)  *Indicate the degree to which each is true* |
| --- |
| The Earth has one big ocean with many features |
| The Earth has always had an ocean |
| The ocean is a major influence on weather and climate |
| The ocean makes Earth hotter* |
| All life arose in the oceans |
| The ocean and humans are interconnected |
| The ocean is largely unexplored |
| **Perceived Knowledge of Marine Science** (1 = Nothing at all to 5 = A great deal (expert-level))  *How much do you know about marine sciences?* |
| **Marine Science Communication Skills** (1 = Strongly Disagree to 5 = Strongly Agree)  *I am confident in my ability to communicate about marine science to my friends and family* |
| **Marine Science Resource Skills** (1 = Strongly Disagree to 5 = Strongly Agree)  *I am confident that I can find resources to help me keep up with learning more about marine science* |
| **Marine Science Stewardship Skills** (1 = Strongly Disagree to 5 = Strongly Agree)  *I am confident that I can engage in stewardship behaviors to help the oceans* |

*Reverse coded variable

Table S4: Survey questions used to determine influences in career and career development.

| **Importance of Marine Science on Career** (1 = Not at all important to 5 = Extremely important)  *Please indicate how important marine science is to your career* |
| --- |
| **Importance of the Program on Graduation** (1 = Strongly Disagree to 5 = Strongly Agree)  *Please indicate your level of agreement with the following statements* |
| Participating in CI made me reconsider my career path to one focused on marine biology |
| Communication skills I learned in CI have helped me in my career |
| Conservation strategies I learned in CI have helped me in my career |
| Research skills I learned in CI have helped me in my career |
| CI assisted in making me more confident in my career field |
| CI assisted in making me more confident in my career field |
| *Cronbach’s alpha = 0.83* |
| **Pursuance of STEM Career** (Medical, Research, other STEM field)  *What is your current job/career field?* |
| Education |
| Medical |
| Research |
| Communication |
| Industry |
| Have not yet graduated |
| Graduate/continuing student (text entry) |
| Other (text entry) |
| \| **Pursuance of Further Education** (Graduate/continuing student, Have not yet graduated)  *What is your current job/career field?* \| \| --- \| \| Education \| \| Medical \| \| Research \| \| Communication \| \| Industry \| \| Have not yet graduated \| \| Graduate/continuing student (text entry) \| \| Other (text entry) \| |

Table S5: Survey questions used to decipher attitudes about science, marine science, and climate change.

| **Science identity and belonging** (1 = Strongly disagree to 5 = Strongly agree)  *Please indicate your level of agreement with the following statements* |
| --- |
| I have a strong sense of belonging to the science community |
| I feel like I belong in the field of science |
| Scientific work is appealing to me |
| It is important to take part in science communication activities with non-science personnel |
| I have a duty to take part in science communication activities targeting the general public |
| I think discussing new theories and ideas about science is important |
| I think it is valuable to conduct research that builds the world's scientific knowledge |
| I think science can solve many of today's world challenges |
| I feel discovering something new in science is thrilling |
| *Cronbach’s alpha = 0.85* |
| **Perception of Climate Change Threat on Marine Environment** (1 = No threat at all to 5 = High threat)  *What level of threat does each of the following topics pose to ocean health?* |
| Ocean warming |
| Plastic (or other trash) pollution and marine debris |
| Ocean acidification (lower pH) |
| Loss of endangered species |
| Nutrient pollution (eutrophication) |
| Overfishing |
| Habitat Loss |
| Diseases and pathogens |
| Importance of Conservation on Daily Life (1 = Not at all important to 5 = Extremely important)  *Please indicate how important conservation is to you in your daily life* |

Table S6. Multiple model comparison of seven multiple regression and one logistical regression models exploring eight dependent variables derived from student surveys. The independent variables included in the model were three fixed demographic covariates (age, gender, ideology) and four independent variables associated with aspects of our creative inquiry program (duration, mentorship, research emphasis, and outreach emphasis). A minimum ΔAICc approach was used to identify the best fit, equally probable models as indicated by gray shading for those with ΔAICc < 2.0. Individual factors significance are indicated in *italics* (P < 0.10), **bold** (P < 0.05) and **bold underlined** (P < 0.01).

|  | Age | Gender | Ideology | Duration | Mentor | Research | Outreach |  |  |  |  |  |
| --- | --- | --- | --- | --- | --- | --- | --- | --- | --- | --- | --- | --- |
| Dependent variable | β1 | β2 | β3 | β4 | β5 | β6 | β7 | F | P | adj R^2^ | AICc | ΔAICc |
|  |  |  |  |  |  |  |  |  |  |  |  |  |
| Perceived Knowledge | 0.0066 | 0.1293 | 0.0395 | *0.1014* | **0.5824** |  |  | 2.8304 | 0.0369 | 0.2337 | 74.954 | 0.000 |
| of Marine Science | -0.0148 | -0.0429 | 0.0473 |  | **0.7002** |  |  | 2.2155 | 0.0951 | 0.1394 | 76.398 | 1.444 |
|  | -0.0110 | 0.0173 | -0.0457 | **0.1250** |  |  |  | 2.0315 | 0.1194 | 0.1209 | 77.059 | 2.105 |
|  | -0.0372 | 0.0427 | 0.0297 |  | **0.6536** | -0.0893 | -0.5693 | 2.0896 | 0.0923 | 0.1789 | 79.507 | 4.553 |
|  | -0.0433 | -0.2347 | -0.0580 |  |  |  |  | 0.6477 | 0.5912 | -0.0365 | 80.236 | 5.282 |
|  | -0.0218 | 0.1531 | 0.0344 | 0.0772 | **0.5919** | 0.0886 | -0.2490 | 2.0085 | 0.0979 | 0.1904 | 81.773 | 6.819 |
|  | -0.0617 | -0.1317 | -0.0703 |  |  | -0.1506 | -0.6539 | 1.1126 | 0.3790 | 0.0184 | 82.631 | 7.677 |
|  | -0.0376 | 0.0414 | -0.0510 | 0.1054 |  | 0.1003 | -0.2055 | 1.3496 | 0.2746 | 0.0653 | 83.523 | 8.569 |
|  |  |  |  |  |  |  |  |  |  |  |  |  |
| Perception of Climate | 0.0270 | 0.1244 | **0.1226** | **0.0515** |  | -0.2261 | 0.1809 | 4.0953 | 0.0057 | 0.3823 | 16.101 | 0.000 |
| Change Threat on the | -0.0109 | 0.1757 | **0.1185** | *0.0349* |  |  |  | 3.7887 | 0.0148 | 0.2710 | 16.673 | 0.572 |
| Marine Environment | -0.0199 | 0.1054 | **0.1150** |  |  |  |  | 3.6885 | 0.0240 | 0.2118 | 17.162 | 1.061 |
|  | 0.0152 | 0.0397 | **0.1132** |  |  | **-0.3488** | -0.0382 | 3.4501 | 0.0166 | 0.2899 | 18.012 | 1.911 |
|  | -0.0158 | 0.1330 | **0.1302** |  | 0.1007 |  |  | 2.9645 | 0.0383 | 0.2075 | 19.262 | 3.161 |
|  | -0.0090 | 0.1879 | **0.1277** | 0.0323 | 0.0631 |  |  | 3.0216 | 0.0287 | 0.2520 | 19.626 | 3.525 |
|  | 0.0285 | 0.1347 | **0.1305** | *0.0489* | 0.0548 | -0.2272 | 0.1768 | 3.4507 | 0.0113 | 0.3637 | 19.725 | 3.624 |
|  | 0.0187 | 0.0648 | **0.1276** |  | 0.0939 | **-0.3399** | -0.0260 | 2.9889 | 0.0252 | 0.2845 | 20.656 | 4.555 |
|  |  |  |  |  |  |  |  |  |  |  |  |  |
|  |  |  |  |  |  |  |  |  |  |  |  |  |
|  |  |  |  |  |  |  |  |  |  |  |  |  |
|  |  |  |  |  |  |  |  |  |  |  |  |  |
| Table S6 continued | Age | Gender | Ideology | Duration | Mentor | Research | Outreach |  |  |  |  |  |
| Dependent variable | β1 | β2 | β3 | β4 | β5 | β6 | β7 | F | P | adj R^2^ | AICc | ΔAICc |
|  |  |  |  |  |  |  |  |  |  |  |  |  |
| Science Identity | **-0.0451** | 0.0071 | 0.0218 | **0.0685** |  |  |  | 4.5380 | 0.0065 | 0.3205 | 37.981 | 0.000 |
| and Belonging | *-0.0420* | 0.0272 | 0.0372 | **0.0642** | 0.1046 |  |  | 3.6502 | 0.0129 | 0.3063 | 40.774 | 2.793 |
|  | **-0.0629** | -0.1310 | 0.0151 |  |  |  |  | 3.3457 | 0.0338 | 0.1899 | 41.498 | 3.517 |
|  | **-0.0556** | -0.0819 | 0.0421 |  | 0.1792 |  |  | 2.8440 | 0.0442 | 0.1974 | 43.143 | 5.162 |
|  | **-0.0696** | -0.8543 | 0.0093 |  |  | -0.0938 | -0.3174 | 2.6133 | 0.0493 | 0.2119 | 44.732 | 6.751 |
|  | -0.0548 | 0.0207 | 0.0209 | *0.0646* |  | 0.0599 | -0.0425 | 2.8403 | 0.0311 | 0.2690 | 44.809 | 6.828 |
|  | *-0.0638* | -0.0441 | 0.0327 |  | 0.1545 | -0.0793 | -0.2974 | 2.3271 | 0.0651 | 0.2097 | 47.227 | 9.246 |
|  | -0.0520 | 0.0409 | 0.0368 | 0.0595 | 0.1069 | 0.0578 | -0.0504 | 2.4439 | 0.0499 | 0.2520 | 48.230 | 10.249 |
|  |  |  |  |  |  |  |  |  |  |  |  |  |
| Importance of Marine | *-0.1258* | -0.1329 | 0.0463 | **0.2164** |  |  |  | 3.8630 | 0.0136 | 0.2762 | 112.135 | 0.000 |
| Science on Career | -0.1082 | -0.0212 | 0.1314 | **0.1929** | 0.5809 |  |  | 3.4084 | 0.0175 | 0.2864 | 113.851 | 1.716 |
|  | **-0.0181** | -0.5694 | -0.0251 |  |  |  |  | 2.7898 | 0.0596 | 0.1518 | 115.125 | 2.990 |
|  | **-0.1489** | -0.3489 | 0.1462 |  | 0.8050 |  |  | 2.8161 | 0.0472 | 0.1949 | 115.437 | 3.302 |
|  | -0.1473 | -0.5223 | 0.0016 |  |  | -0.8821 | *-1.1310* | 2.4421 | 0.0621 | 0.1937 | 117.635 | 5.500 |
|  | -0.1064 | -0.2294 | 0.0342 | 0.1783 |  | -0.4575 | -0.3726 | 2.4675 | 0.0530 | 0.2269 | 118.745 | 6.610 |
|  | -0.1206 | -0.3324 | 0.1105 |  | 0.7110 | -0.8154 | -1.0391 | 2.4388 | 0.0553 | 0.2234 | 118.883 | 6.748 |
|  | -0.0907 | -0.1178 | 0.1196 | 0.1501 | 0.5909 | -0.4692 | -0.4160 | 2.3325 | 0.0592 | 0.2371 | 121.037 | 8.902 |
|  |  |  |  |  |  |  |  |  |  |  |  |  |
| Marine Science | 0.0189 | -0.0075 | 0.0570 | *0.0785* | **0.7357** |  |  | 3.8940 | 0.0095 | 0.3253 | 67.982 | 0.000 |
| Communication Skills | 0.0023 | -0.1409 | 0.0630 |  | **0.8269** |  |  | 3.7663 | 0.0152 | 0.2694 | 68.297 | 0.315 |
|  | -0.0438 | -0.0174 | 0.0514 |  | **0.7959** | 0.2097 | -0.4263 | 3.3223 | 0.0159 | 0.3171 | 70.768 | 2.786 |
|  | -0.0310 | 0.0741 | 0.0553 | 0.0641 | **0.7447** | 0.3575 | -0.1603 | 3.0483 | 0.0202 | 0.3233 | 73.191 | 5.209 |
|  | -0.0032 | -0.1490 | -0.0507 | **0.1083** |  |  |  | 1.8424 | 0.1510 | 0.1009 | 74.730 | 6.748 |
|  | -0.0313 | -0.0367 | -0.0613 |  |  |  |  | 0.7743 | 0.5185 | -0.0230 | 76.908 | 8.926 |
|  | -0.0736 | -0.2300 | -0.0704 |  |  | 0.1351 | -0.5292 | 1.1755 | 0.3489 | 0.0284 | 79.290 | 11.308 |
|  | -0.0508 | -0.0664 | -0.0522 | 0.0996 |  | 0.3722 | -0.1056 | 1.4016 | 0.2547 | 0.0743 | 80.199 | 12.217 |
|  |  |  |  |  |  |  |  |  |  |  |  |  |
|  |  |  |  |  |  |  |  |  |  |  |  |  |
| Table S6 continued | Age | Gender | Ideology | Duration | Mentor | Research | Outreach |  |  |  |  |  |
| Dependent variable | β1 | β2 | β3 | β4 | β5 | β6 | β7 | F | P | adj R^2^ | AICc | ΔAICc |
|  |  |  |  |  |  |  |  |  |  |  |  |  |
| Importance of the | -0.0468 | 0.4967 | 0.1511 |  | **0.6629** |  |  | 3.1520 | 0.0307 | 0.2230 | 72.107 | 0.000 |
| Program After | -0.0382 | 0.5661 | 0.1479 | 0.4080 | **0.6155** |  |  | 2.6290 | 0.0482 | 0.2136 | 74.634 | 2.527 |
| Graduation | *-0.0738* | 0.3151 | 0.0513 |  |  |  |  | 1.6227 | 0.2073 | 0.0586 | 76.127 | 4.020 |
|  | -0.0568 | 0.4477 | 0.0577 | 0.0657 |  |  |  | 1.6438 | 0.1933 | 0.0790 | 77.377 | 5.270 |
|  | -0.0080 | 0.4257 | 0.1456 |  | **0.6488** | -0.4207 | -0.1044 | 2.2028 | 0.0781 | 0.1939 | 77.811 | 5.704 |
|  | 0.0018 | 0.4975 | 0.1487 | 0.0497 | **0.6090** | -0.3060 | 0.1020 | 1.9358 | 0.1097 | 0.1792 | 81.078 | 8.971 |
|  | -0.0323 | 0.2524 | 0.0463 |  |  | -0.4815 | -0.1883 | 1.1769 | 0.3483 | 0.0286 | 81.183 | 9.076 |
|  | -0.0143 | 0.3818 | 0.0600 | 0.0788 |  | -0.2940 | 0.1467 | 1.2030 | 0.3386 | 0.0390 | 83.260 | 11.153 |
|  |  |  |  |  |  |  |  |  |  |  |  |  |
| Logistic Regression | Age | Gender | Ideology | Duration | Mentor | Research | Outreach |  |  |  |  |  |
| Dependent variable | β1 | β2 | β3 | β4 | β5 | β6 | β7 | χ^2^ | P |  | AICc | ΔAICc |
|  |  |  |  |  |  |  |  |  |  |  |  |  |
| Pursuance of | *1.0985* | 1.2829 | 0.1430 | *-0.4276* |  |  |  | 18.6570 | 0.0009 |  | 35.122 | 0.000 |
| Further Education | *1.3261* | 1.8268 | 0.6023 | **-0.5447** | 2.3397 |  |  | 20.8549 | 0.0009 |  | 36.026 | 0.904 |
|  | **0.7826** | 1.8452 | 0.2492 |  |  |  |  | 14.3409 | 0.0025 |  | 36.578 | 1.456 |
|  | **0.8194** | 2.0659 | 0.3814 |  | 0.7227 |  |  | 14.6906 | 0.0054 |  | 39.090 | 3.968 |
|  | 0.7165 | 1.6758 | 0.2533 |  |  | 1.3532 | 1.8076 | 17.2945 | 0.0040 |  | 39.586 | 4.464 |
|  | 1.0588 | 1.2972 | 0.1491 | -0.3903 |  | 0.1056 | 0.2360 | 18.6780 | 0.0047 |  | 41.572 | 6.450 |
|  | *0.8164* | 2.0091 | 0.4771 |  | 1.3080 | 1.4323 | *2.0499* | 18.1835 | 0.0058 |  | 42.067 | 6.945 |
|  | *1.4208* | 1.7488 | 0.6196 | -0.6050 | 2.4093 | -0.4987 | -0.3301 | 20.8970 | 0.0039 |  | 43.028 | 7.906 |

Table S7. Demographic information related to sex and race/ethnicity across the university level, major level (demographics across most majors (~95% of students) who actively participate either program including Animal Veterinary Sciences, Environmental and Natural Resource Sciences – Conservation Biology, and Biological Sciences), program level, and survey level. Upper-level data was retrieved from Clemson University’s 2020 open-access enrollment data. University and major demographics were extremely similar throughout all years of participation.

| **Gender** | **University** | **Majors** | **Program** | **Survey** |
| --- | --- | --- | --- | --- |
| *Male* | 50.6% | 29.4% | 20.5% | 14.7% |
| *Female* | 49.4% | 70.6% | 79.5% | 85.3% |
| **Race/Ethnicity** | **University** | **Majors** | **Program** | **Survey** |
| *White* | 80.2% | 78.6% | 88.5% | 88.6% |
| *Hispanic* | 6.0% | 5.9% | 2.6% | 2.9% |
| *Black* | 5.8% | 7.3% | 3.8% | 2.9% |
| *Asian* | 2.7% | 3.3% | 3.8% | 2.9% |
| *Native* | 0.3% | 0.4% | 0.6% | 0.0% |
| *Other* | 5.0% | 4.5% | 0.6% | 2.9% |
| **Total Individuals** | 20,878 | 2,165 | 156 | 35 |

Table S8. All quotes from the survey responses to: “If you have comments on your experience with the Creative Inquiries or this survey, please let us know below.”

| **Responses** |
| --- |
| My entire career path was transformed because of my experiences in these CIs. I can't overstate the effect they've had on my life. |
| Creative inquiry was the most valuable part of my Clemson experience. Without CI, I would have next to nothing positive to say about Clemson bar the environment. CI allowed me to experience the research process for myself, from start to finish. It taught me so much about the practical applications of research and scientific work that I never learned in class. It was also an incredible boon to my personal life, as it was the only place I met like-minded people to socialize with and whom I enjoyed working with. I was fortunate to take part in two CIs with this lab, both of which gave me unique and priceless experiences that I will forever be grateful for. |
| Without CI, I wouldn't have been prepared for graduate school the way that I am! I miss being apart of this great program but I am so appreciative of the support and training you all provided for me. |
| I participated in Creative Inquiry during the Fall 2009, Fall 2010, and Spring 2011 semesters, but I was only able to select the Spring 2011. Additionally, I graduated in 2011, but the earliest year I was able to select in the survey was 2012. |
| I did not put all of the correct semesters for my time working in the CI. I was in the lab for Spring 2019, Summer 2019, Fall 2019 and helped some during Spring 2020. Sorry for the inconvenience this year has made me lose track of time. |
| I had a wonderful experience in this creative inquiry! I loved watering my love and appreciation for the ocean and its wildlife through this research experience. |
| I don't remember what semester/semesters I participated in CMR, but I graduated from Clemson in May 2010. |
| I actually graduated in 2011 and participated in CMR from 2009-2011 (2011 graduation wasn't an option on this survey). When I look back on my time at Clemson, CMR was one of the most worthwhile things I participated in. It taught me so much about not only the research process, but also what I was capable of. I love telling people about my blue crab research, my trip to the keys, and my presentations at the Benthic Ecology meetings. Dr Childress was one of those professors that truly cared about his students and I'm glad to see he has continued with CI. |
| Loved it! So glad to be a part of it! Very beneficial as an undergrad and staging involved as a graduate student ! |
| I loved this experience in college but also was super low on the totem pole. I got to hand feed crabs and lobsters, always wanted to be more involved but was not a master's track for con bio. |
| This CI was the best part of my undergrad career and the most enjoyable. A lot of times people don’t think that marine and environmental science apply to the medical/public health field but I’ve been able to be an advocate for how they impact each other including through climate extremities (natural disasters), environmental changes impacting housing and food availability, and pollution/toxins negatively impacting health. This CI also improved my research skills, communication skills, and professionalism. |
| It was a great experience, I would totally do it again if I could! |
